# The divergent vertical pattern and assembly of soil bacterial and fungal communities in response to short-term warming in an alpine peatland

**DOI:** 10.3389/fpls.2022.986034

**Published:** 2022-09-08

**Authors:** Xiaodong Wang, Yong Li, Zhongqing Yan, Yanbin Hao, Enze Kang, Xiaodong Zhang, Meng Li, Kerou Zhang, Liang Yan, Ao Yang, Yuechuan Niu, Xiaoming Kang

**Affiliations:** ^1^Wetland Research Center, Institute of Ecological Conservation and Restoration, Chinese Academy of Forestry, Beijing, China; ^2^Beijing Key Laboratory of Wetland Services and Restoration, Beijing, China; ^3^Sichuan Zoige Wetland Ecosystem Research Station, Tibetan Autonomous Prefecture of Aba, Aba, China; ^4^College of Life Sciences, University of Chinese Academy of Sciences, Beijing, China

**Keywords:** soil microbial community, alpine peatland, community assembly, vertical structure, warming

## Abstract

Soil microbial communities are crucial in ecosystem-level decomposition and nutrient cycling processes and are sensitive to climate change in peatlands. However, the response of the vertical distribution of microbial communities to warming remains unclear in the alpine peatland. In this study, we examined the effects of warming on the vertical pattern and assembly of soil bacterial and fungal communities across three soil layers (0–10, 10–20, and 20–30 cm) in the Zoige alpine peatland under a warming treatment. Our results showed that short-term warming had no significant effects on the alpha diversity of either the bacterial or the fungal community. Although the bacterial community in the lower layers became more similar as soil temperature increased, the difference in the vertical structure of the bacterial community among different treatments was not significant. In contrast, the vertical structure of the fungal community was significantly affected by warming. The main ecological process driving the vertical assembly of the bacterial community was the niche-based process in all treatments, while soil carbon and nutrients were the main driving factors. The vertical structure of the fungal community was driven by a dispersal-based process in control plots, while the niche and dispersal processes jointly regulated the fungal communities in the warming plots. Plant biomass was significantly related to the vertical structure of the fungal community under the warming treatments. The variation in pH was significantly correlated with the assembly of the bacterial community, while soil water content, microbial biomass carbon/microbial biomass phosphorous (MBC/MBP), and microbial biomass nitrogen/ microbial biomass phosphorous (MBN/MBP) were significantly correlated with the assembly of the fungal community. These results indicate that the vertical structure and assembly of the soil bacterial and fungal communities responded differently to warming and could provide a potential mechanism of microbial community assembly in the alpine peatland in response to warming.

## Introduction

The greenhouse effect has been increasing over the past few decades due to the cumulative impact of human activities, leading to global warming (Zandalinas et al., 2021). The global average surface temperature in the first 20 years of the twenty-first century (2001–2020) has increased by 0.99 (0.84–1.10)°C compared with that during 1850–1900 (IPCC, 2021). Peatlands cover only 3% of the Earth's land surface but contain 1,055 Gt of soil carbon (Nichols and Peteet, 2019), which is roughly equivalent to 45% of global soil C (Nichols and Peteet, 2019; Xue D. et al., 2021). Peatlands are a major contributor to global greenhouse gas emissions (Tiemeyer et al., 2016) with a function of buffering the effects of climate warming (Frolking and Roulet, 2007). However, peatlands are suffering massive degradation under climate change and human disturbance, which is affecting the global emissions of greenhouse gases (Chen et al., 2013). Thus, it is necessary to clarify the effects of warming on peatlands.

Warming accelerates carbon emission from subsurface peat, leading to a decreased peatland carbon sink (Dorrepaal et al., 2009; Helbig et al., 2022). The alpine peatlands are more sensitive to warming due to their high altitude (Mclaughlin and Webster, 2018; Zhang et al., 2022). Previous studies have shown that climate warming strongly affects the alpine ecosystem microbial communities on the Qinghai-Tibet Plateau (Zhao et al., 2014; Liu et al., 2016; Zhang K. et al., 2016; Kang et al., 2022). Soil microbes play a vital role in biogeochemical processes and other ecosystem-level decomposition and nutrient cycling processes in peatland and the repose of the microbial community to warming enhances the temperature sensitivity of soil respiration (Bardgett and Van Der Putten, 2014; Anthony et al., 2020). Warming has altered the microbial biomass, community composition, community succession, and network complexity and stability (Blankinship et al., 2011; Guo et al., 2018, 2019; Yuan et al., 2021). However, warming affects the soil bacterial and fungal communities differently. Bacterial communities are more sensitive than fungal communities in topsoil (Guo et al., 2019; De Oliveira et al., 2020; Kanzaki and Takemoto, 2021). Moreover, previous studies focused on the horizontal structure of the soil microbial communities in different soil layers (Du et al., 2017; Jiao et al., 2018; Chen et al., 2020), and the alpha diversity of different microbial taxa varied by depth (Jiao et al., 2018). Soil pH has been reported to be the driving factor for the horizontal structure of the bacterial community (Xia et al., 2016; Liu et al., 2018; Kang et al., 2021). More dimensions must be considered when studying soil microbial communities because of the spatial heterogeneity of the soil and the three-dimensional distribution of microbiomes in soil (Xue R. et al., 2021). However, the vertical responses of soil bacterial and fungal communities to warming remain unclear.

Investigating the ecological processes driving community assembly contributes to disentangling the mechanisms of the microbial communities in response to climate change (Ponisio et al., 2019). Traditional niche theory hypothesizes that community structures are dominated by deterministic factors, such as environmental conditions and the interactions between species, which are referred to as deterministic processes (Chesson, 2000; Fargione et al., 2003; Kraft et al., 2014). In contrast, the neutral theory holds that community structures are determined by stochastic processes, such as birth, death, extinction, speciation, and colonization (Hubbell, 2001; Chave, 2004). The deterministic process and the stochastic process have been recently determined to jointly regulate community assembly (Chase, 2010; Chase and Myers, 2011; Stegen et al., 2016; Cai et al., 2020), but their relative importance in driving community assembly has not been determined (Vellend et al., 2014; Zhou et al., 2014; Tonkin et al., 2018). The dispersal-niche continuum index (DNCI), a standardized effect-size index, has been used to compare the predominance of niche-based vs. dispersal-based processes between multiple datasets (Vilmi et al., 2020), which has a potential ability to reveal the relative importance of ecological processes across soil layers.

The temperature of the Zoige alpine peatland, which is one of the largest and highest plateau peatlands (Chen et al., 2014) in the northeastern part of the Qinghai-Tibet Plateau, has increased significantly (average 0.4°C per decade) (Yang et al., 2014). To investigate how short-term warming affects the vertical distribution and assembly of the soil bacterial and fungal communities, we initiated a field manipulative experiment in the Zoige alpine peatland. We hypothesize that short-term warming has no effects on the diversity of bacterial and fungal communities (hypothesis I); the vertical distribution of bacterial and fungal communities are both altered after short-term warming (hypothesis II); the assembly processes of the bacterial and fungal communities across soil layers are affected by short-term warming (hypothesis III).

## Materials and methods

### Study area and experimental design

This experiment was conducted at the Axi Ranch on the Zoige National Wetland Natural Reserve (33°47′56″ N, 102°57′28″ E), which is the largest plateau peat bog in the world. The study region has a typical plateau monsoon climate. The annual average temperature ranges from −1 to 3.3°C, and the annual average precipitation is 650–750 mm. The region has a long frost period (October–April) and a short growing season (May–September) (Yan et al., 2021). The dominant plant species are *Blysmus sinocompressus, Carex meyeriana, Carex muliensis, Carex secbrirostris, Eriophorum gracile*, and *Koeleria tibetica*.

The warming experiment was initiated in June 2021, using a completely random design. Two levels of warming treatments (slight warming, Ws; high warming, Wh) and a control (CK) were set up with three replicates. The warming treatments were carried out in open-top chambers consisting of six transparent acrylic isosceles trapezoidal plates with light transmittance > 95%. Temperatures measured on average every 10 days in the 0–10 cm soil layer over the entire growing season (June–September) rose by 0.9 and 1.8°C on average in the Ws and Wh treatments, respectively compared to CK (Supplementary Figure 1).

### Plant and soil sampling

The soil and the above-ground plant biomass were sampled at the end of the growing season in late September 2021 after one growing-season warming treatment. Plant biomass was collected using a square frame (0.5 × 0.5 m) and dried at 65°C for 72 hours before being weighed. Five soil cores (5 cm diameter) were randomly collected at depths of 0–10 cm (up), 10–20 cm (mid), and 20–30 cm (low) from each plot. Then, samples from the same depth at each plot were mixed to form a composite sample. Twenty-seven soil samples (3 treatments ×3 layers ×3 replicates) were taken in total, placed on dry ice, and delivered by express mail to the laboratory in Beijing, China. A subsample of soil from each sample was immediately frozen at −20°C for microbial community analyses and the other subsamples were used to determine the soil physical and chemical indicators.

### Soil physicochemical characteristics

A soil subsample was air-dried, finely ground, and passed through a 0.15 mm sieve to measure soil organic carbon (SOC), dissolved organic carbon (DOC), soil pH, total nitrogen (TN), total phosphorus (TP), available phosphorus (AP), ammonium (${\text{NH}}_{4}^{+}$), nitrate (${\text{NO}}_{3}^{-}$), microbial biomass carbon (MBC), microbial biomass nitrogen (MBN), and microbial biomass phosphorus (MBP), while another sample was passed through a 2 mm sieve with the roots removed to determine soil water content (SWC). Soil pH was assessed using a pH electrode (PB-10, Sartorius, Germany) in a 1:2.5 soil/water solution. SWC was determined using the oven-drying method. SOC was determined by the rapid dichromate oxidation-titration method. DOC was measured on a total organic C analyzer (Vario TOC Cube, Elementar, Germany). Soil TN was determined full-automatic Kjeldahl apparatus (KJELTEC 8400, FOSS, Danmark), and soil TP was determined by spectrophotometer (TAS-990, Persee, Beijing, China) using the method of Wu et al. (2017). A spectrophotometer was used to assess soil AP by molybdenum blue colorimetry (TAS-990, Persee, Beijing, China). ${\text{NH}}_{4}^{+}$ and ${\text{NO}}_{3}^{-}$ concentrations were determined by the extracts of the unfumigated soils using a flow injection auto-analyzer (SANplus, Skalar, Netherlands). The MBC, MBN, and MBP contents were evaluated using the chloroform fumigation extraction method (Brookes et al., 1982, 1985; Vance et al., 1987).

### DNA extraction and polymerase chain reaction (PCR) amplification

Microbial community genomic DNA was extracted using the FastDNA^®^ SPIN Kit for Soil (MP Biomedical, Irvine, CA, USA) according to the manufacturer's instructions. The DNA extract was checked by 1% agarose gel electrophoresis, and DNA concentration and purity were determined with the NanoDrop 2000 UV-vis spectrophotometer (Thermo Scientific, Wilmington, DE, USA). The hypervariable V4 region of the bacterial 16S rRNA gene was amplified with the primer pairs 515F (5′-GTGCCAGCMGCCGCGG-3′) and 806R (5′-GGACTACNVGGGTWTCT-3′), while the fungal ITS gene was amplified with the primers ITS1F (5′ CTTGGTCATTTAGAGGAAGTAA-3′) and ITS2R (5′-GCTGCGTTCTTCATCGATGC-3′) using the PCR thermocycler (GeneAmp^®^ 9700, ABI, Thermo Fisher). The reverse primer was combined with the adapter and barcode sequences for multiplexing, and amplification was performed in 20 μl reaction volumes containing 2 μl of 10 × *TransStart* FastPfu Buffer, 0.2 μl of FastPfu Polymerase, 0.8 mM of each primer (5 μM), 2 μl of 2.5 mM dNTPs, 0.2 μl of BSA, and 10 ng of template DNA. The PCR program consisted of 30 cycles of initial denaturation at 95°C for 3 min, 95°C for 30 s, 55°C for 30 s, 72°C for 45 s, and a final extension at 72°C for 10 min.

Purified amplicons were sequenced in equimolar concentrations and pair-end read on the Illumina MiSeq PE300 platform/NovaSeq PE250 platform (Illumina, San Diego, CA, USA) according to the standard protocol of Majorbio Bio-Pharm Technology Co. Ltd. (Shanghai, China).

### Bioinformatical analyses

The sequenced reads were demultiplexed, quality-filtered using fastp version 0.20.0 (Chen et al., 2018), and merged with FLASH version 1.2.7 (Magoc and Salzberg, 2011) using the following criteria: (i) only overlapping sequences > 10 bp were assembled according to their overlapped sequence. The maximum mismatch ratio of the overlapping region was 0.2. Reads that could not be assembled were discarded; (ii) Samples were distinguished according to the barcode and primers, and the sequence direction was adjusted using exact barcode matching and 2 nucleotide mismatches for primer matching; (iii) the 300 bp reads were truncated at any site receiving an average quality score <20 over a 50 bp sliding window, and truncated reads <50 bp were discarded; reads containing ambiguous characters were also discarded.

UPARSE version 7.1 (Edgar, 2013) was used to cluster the operational taxonomic units (OTUs) with a 97% similarity cutoff (Stackebrandt and Goebel, 1994; Edgar, 2013) and chimeric sequences were identified and removed. The Ribosomal Database Project classifier (version 2.2) with database Silva v138 and UNITE version 10.05.2021 for bacteria and fungi respectively (http://rdp.cme.msu.edu) was used to assign OTU representative sequences at a 70% threshold (Wang et al., 2007; Nilsson et al., 2018).

### Statistical analyses

To make abundances comparable between the samples, the *rrarefy* function in the R package “vegan” (The R Foundation for Statistical Computing, Vienna, Austria) was applied. The effects of treatments and soil layers on soil characters and alpha diversity of the bacteria and fungi (richness, Shannon index, and Pielou's evenness) were tested by two-way nested analysis of variance (ANOVA) (soil layers nested in the treatments), followed by multiple comparisons using the LSD method for the treatments and the soil layers. One-way ANOVA was applied to test the effects of the warming treatments on plant biomass. Bray–Curtis metrics were calculated to determine the dissimilarities in the microbial communities at the taxonomic level across soil layers (*vegdist* function in R package “vegan”). Non-metric multidimensional scaling analysis was conducted to visualize distances between communities with the Bray–Curtis dissimilarity measurements (*metaMDS* function in R package “vegan”). Permutational multivariate analysis of variance (PERMANOVA) and ANOSIM was conducted (*adonis* and *anosim* function in R package “vegan”) to assess the effects of the treatments and soil layers on the taxonomic composition of the microbial communities. The Mantel test was performed to reveal the relationship between the microbial communities and the environmental variables (*mantel* function in R package “vegan”).

The dispersal–niche continuum index (DNCI) was calculated between soil layers and the entire microbial community across soil layers to reveal the ecological processes driving the microbial community across soil layers under the different treatments. Significant positive DNCI values indicate that the community is driven predominantly by the niche process, whereas significant negative values indicate a dispersal-dominated ecological process. If the DNCI distribution does not significantly differ from 0, the dispersal and niche processes were assumed to contribute equally to the community. DNCI analyses were carried out using the R package “DNCImper” available on Github (Vilmi et al., 2020) with 1,000 permutations. Moreover, for each variable (e.g., soil pH), the variation (e.g. |SWCa–SWCb|, where a and b represent samples) and the mean (e.g. [SWCa + SWCb]/2) of each pair of samples was used to calculate Pearson's correlation with the DNCI.

## Results

### Vertical distribution and variation of environmental variables

In addition to soil SWC, MBC/MBP and MBN/MBP were significantly affected by warming (*p* < 0.05). The majority of the soil characters (SWC, SOC, TN, TP, AP, DOC, and ${\text{NH}}_{4}^{+}$) differed more significantly between soil layers (*p* < 0.05) (Table 1). SWC in the 0–10 cm soil layer decreased by 8.36% and 12.86% in the Ws (*p* > 0.05) and Wh (*p* < 0.05) plots, respectively, compared to CK. Soil ${\text{NO}}_{3}^{-}$ concentrations decreased significantly (*p* < 0.05) by 66.55% and 57.10% in the Ws and Wh plots, respectively. Neither Ws nor Wh had significant effects on other soil characters. None of the soil characters in the mid (10–20 cm) or lower (20–30 cm) soil layers were significantly affected by the warming treatments. Plant biomass was not significantly affected by the warming treatments (*p* > 0.05) (Table 1).

**Table 1:** Results of two-way nested ANOVAs for the effects of treatment and soil depth (nested within treatments) on soil characters and the results of one-way ANOVA for plant biomass affected by the treatments.

	**Treatments**		**Layers**	
	***F***	**Pr (>F)**	***F***	**Pr (>F)**
SWC	5.011	0.019*	4.830	0.004**
pH	0.880	0.432	2.308	0.079
SOC	0.074	0.929	14.879	0.001***
TN	0.024	0.976	8.724	0.001***
TP	0.741	0.490	4.892	0.004**
AP	0.278	0.760	4.779	0.004**
DOC	0.153	0.859	10.002	0.001***
${\text{NH}}_{4}^{+}$	0.056	0.946	3.824	0.012*
${\text{NO}}_{3}^{-}$	2.255	0.134	2.467	0.064
MBC	2.015	0.162	6.938	0.001**
MBN	0.177	0.839	5.493	0.002**
MBP	1.416	0.268	4.322	0.007**
MBC/MBN	1.088	0.358	2.744	0.045*
MBC/MBP	3.906	0.039*	2.046	0.112
MBN/MBP	4.555	0.025*	1.780	0.160
Biomass	3.383	0.104	/	/

MBC, microbial biomass carbon; MBN, microbial biomass nitrogen; MBP, microbial biomass phosphorous; Biomass, plant biomass; *0.01 < *p* < 0.05; **0.001 < *p* < 0.01; ***p < 0.001.

### Composition of soil bacterial and fungal communities

Approximately 1,365,171 and 1,562,875 sequences (27 samples, average 50,562 and 57,884 sequences) were obtained for the bacterial and fungal communities, respectively. A total of 11,108 bacterial and 3,693 fungi OTUs were obtained at the 97% similarity level based on these sequences.

**Figure 1:**
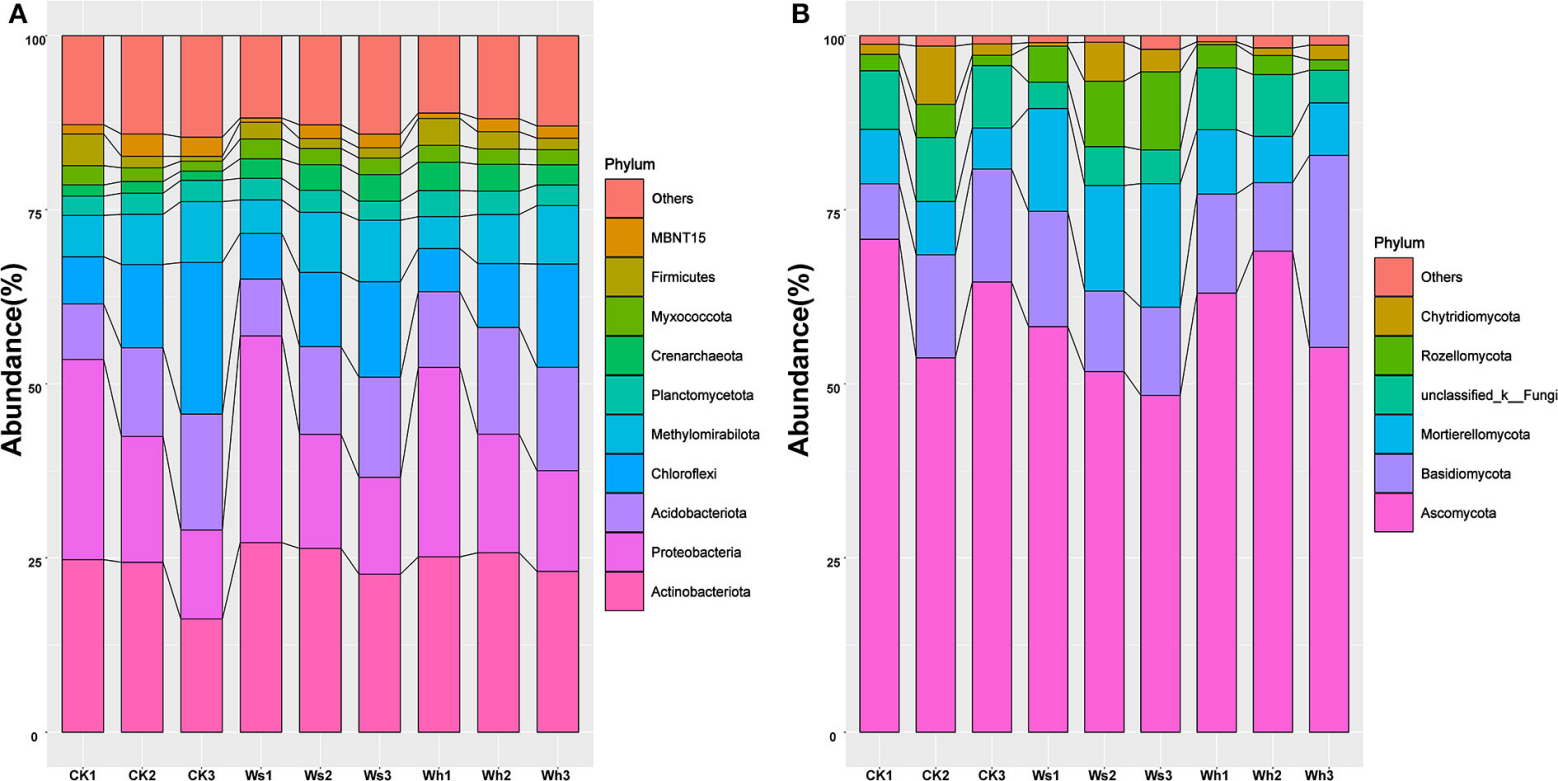
Relative abundance of the dominant bacteria **(A)** and fungi **(B)** groups at phylum level at different soil layers. Ws, slight warming; Wh, high warming; CK1, 0–10 cm depth in control plots; CK2, 10–20 cm depth in control plots; CK3, 20–30 cm depth in control plots.

The dominant groups (>10%) in the bacterial community were Proteobacteria and Actinobacteriota, which were 28.57 and 25.69% of the total (Figure 1A, Supplementary Figure 2), respectively. The relative abundances of Actinobacteriota, Crenarchaeota, Firmicutes, MBNT15, and Verrucomicrobiota were affected by the warming treatments, and the majority of the groups differed significantly (*p* < 0.05) in the soil layers (Supplementary Figure 3). Ascomycota (64.02%), Basidiomycota (12.91%), and Mortierellomycota (10.59%) were the dominant fungal groups (Figure 1B, Supplementary Figure 2). The relative abundance of Mortierellomycota was significantly different between the treatments but no significant effects were observed between the soil layers of the other groups (Supplementary Figure 4).

### Alpha and beta diversity of soil bacterial and fungal communities

**Figure 2:**
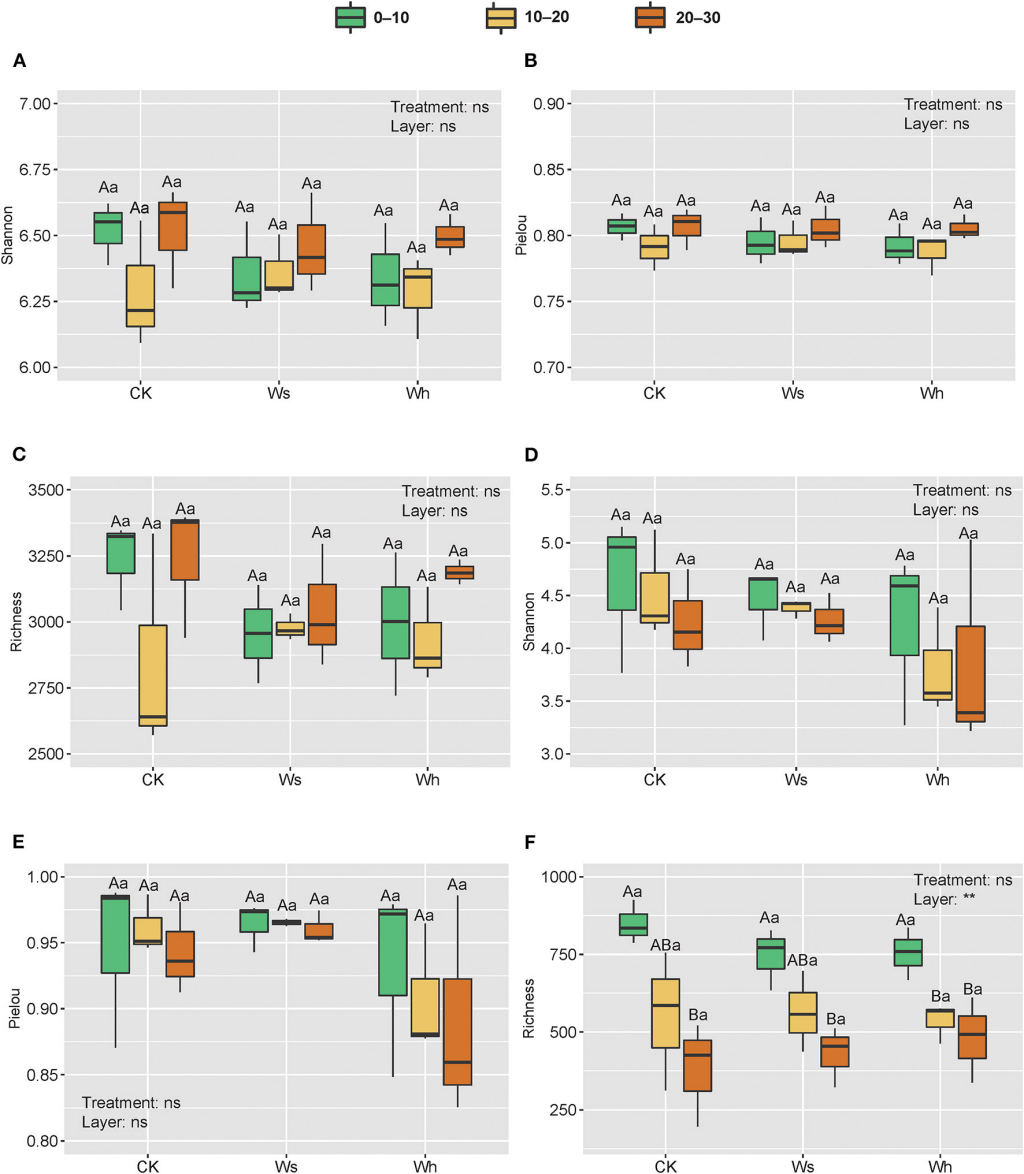
Richness and alpha diversity index of soil bacterial **(A–C)** and fungal **(D–F)** communities. Ws, slight warming; Wh, high warming. ** and ns indicate the significant levels for treatments and soil depths (nested within treatments) at 0.01 and non-significant, respectively. Boxplots not sharing a common capital letter are significantly different (*p* < 0.05) among soil layers while different small letters represent significantly different (*p* < 0.05) among treatments.

OTU richness, the Shannon diversity index, and Pielou's evenness were used as indicators of alpha diversity. The results of two-way nested ANOVA showed that the warming treatments had no significant effects on alpha diversity of either the bacterial or the fungal community (*p* > 0.05) but the soil layers had significant effects on richness (*p* < 0.01) of the fungal community (Figure 2).

**Figure 3:**
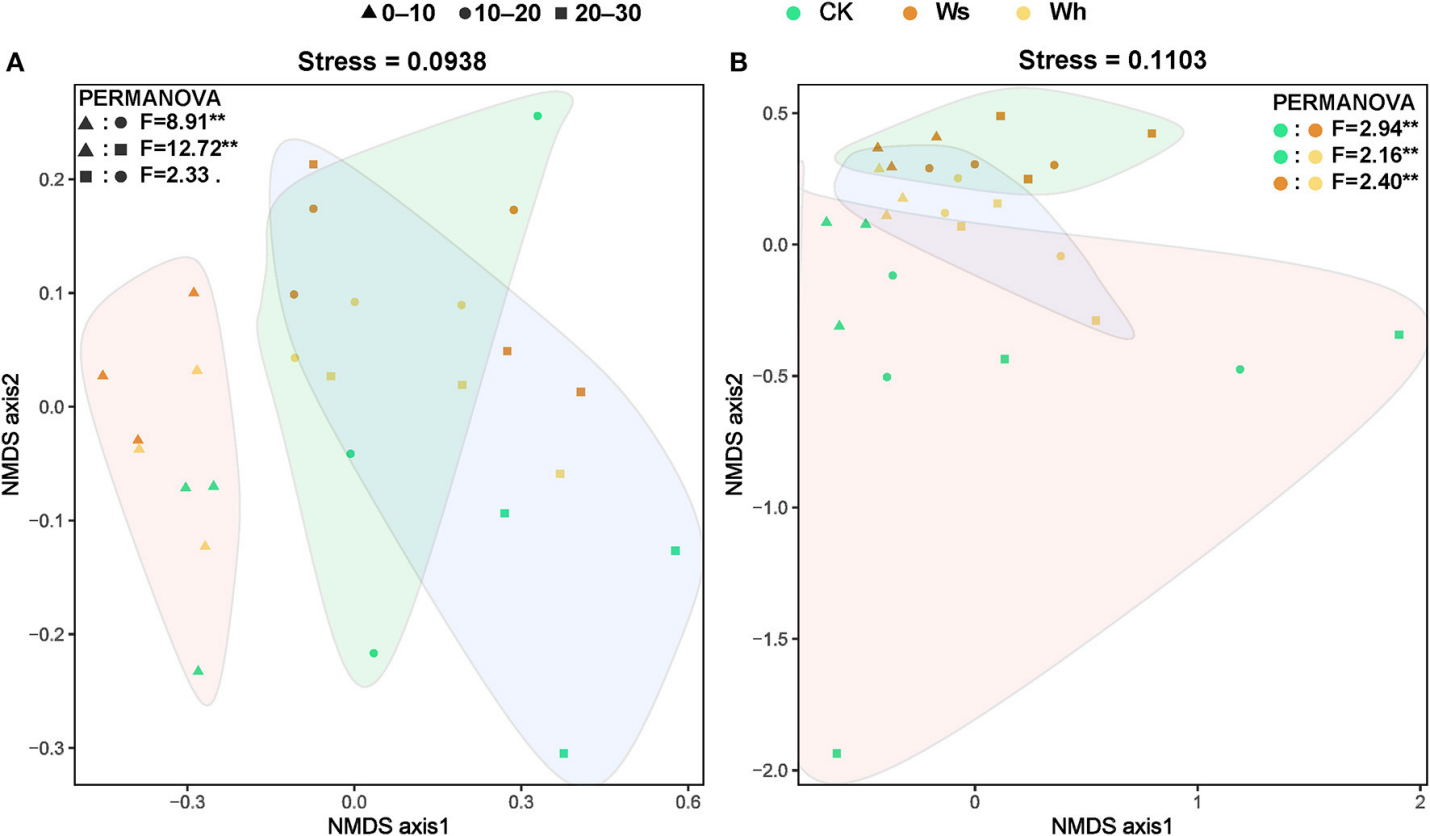
Non-metric multidimensional scaling (NMDS) ordination of the soil bacterial **(A)** and fungal **(B)** community structure based on the Bray-Curtis distance. The filled graphs cover significantly different groups of bacteria and fungi communities by PERMANOVA. Ws, slight warming; Wh, high warming; **0.001 < *p* < 0.01.

The Bray-Curtis index for the bacterial communities between mid and low soil layers decreased with temperature, and the dissimilarity of the bacterial communities increased with distance (Supplementary Figure 5A). No significant differences in the Bray-Curtis index of the fungal communities were observed between the treatments or by distance (Supplementary Figure 5B). The results of PERMANOVA and ANOSIM revealed a significant difference between the upper layer (0–10 cm) and the mid (10–20 cm) or lower (20–30) layer bacterial communities, while no significant difference was detected between the mid and lower layers (Figure 3A, Supplementary Tables 2, 3). The fungal communities were significantly different (*p* < 0.01) under the different treatments (Figure 3B). However, the bacterial communities under the different treatments and the fungal communities at different soil depths were not significantly different (Figure 3).

### Ecological processes and factors influencing the microbial communities

**Figure 4:**
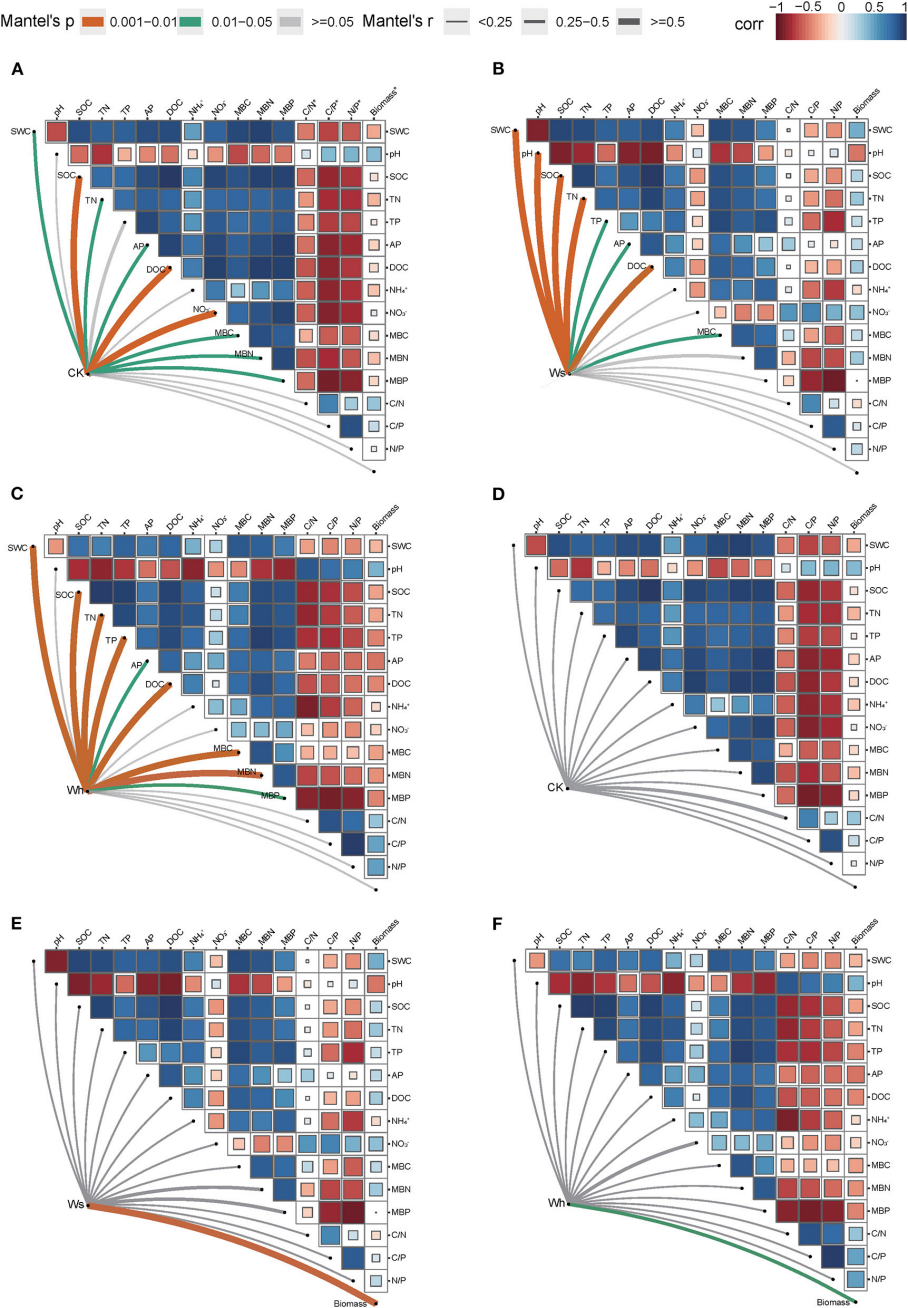
Mantel tests between the vertical structure of soil bacterial **(A–C)** and fungal **(D–F)** communities with environmental factors. Ws, slight warming; Wh, high warming; MBC, microbial biomass carbon; MBN, microbial biomass nitrogen; MBP, microbial biomass phosphorous; C/N*, MBC/MBN; C/P*, MBC/MBP; N/P*, MBN/MBP; Biomass*, plant biomass.

**Figure 5:**
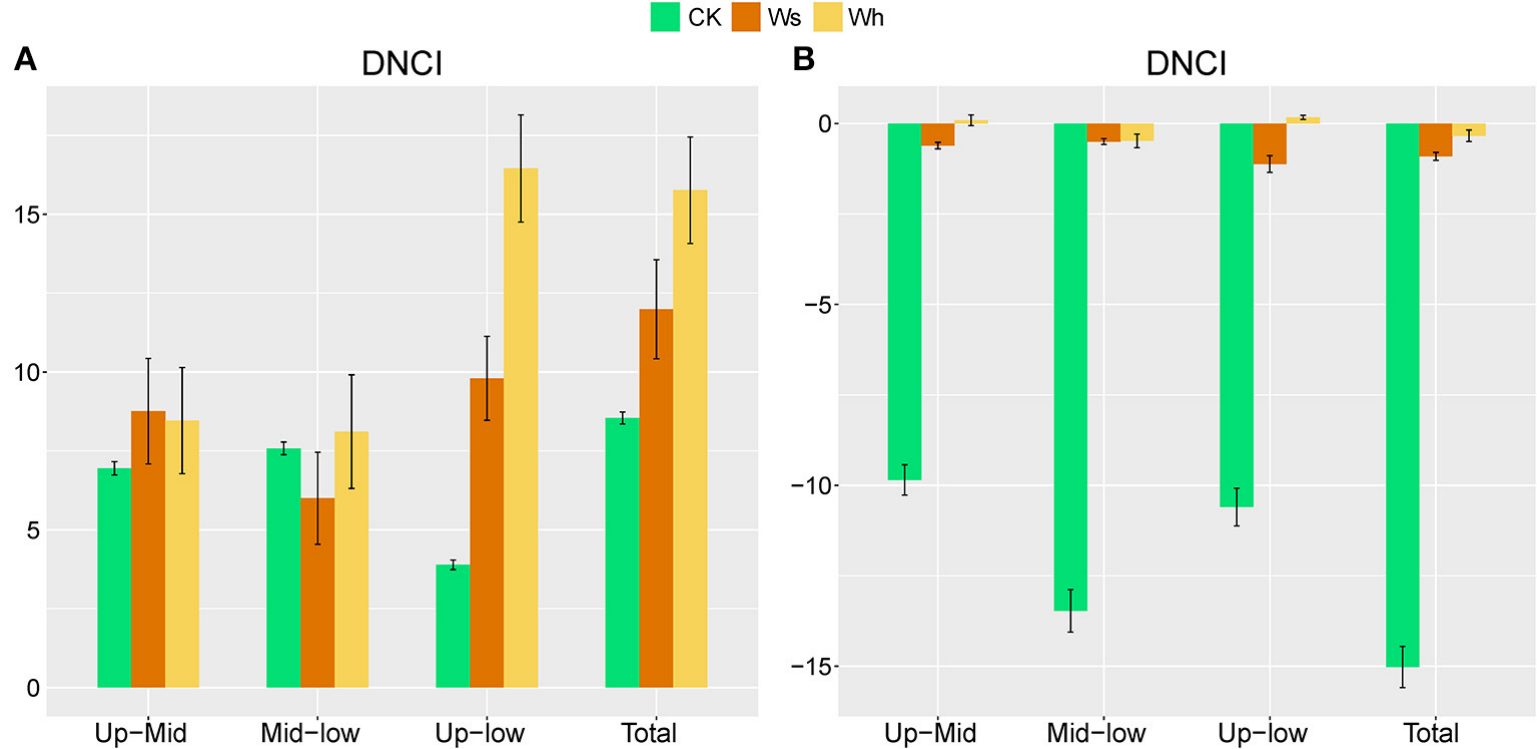
Dispersal–niche continuum index (DNCI) of soil bacterial **(A)** and fungal **(B)** communities across soil layers. Means ± standard errors are shown in the figures. The main assembly process is either dispersal (DNCI <0), niche (DNCI>0), or dispersal–niche (DNCI≈0). Ws, slight warming; Wh, high warming. Up, 0–10 cm; Mid, 10–20 cm; Low, 20–30 cm.

In the CK plots, SOC, DOC, NO3-, SWC, TN, AP, MBC, MBN and MBP were the important factors contributing to variation in soil bacterial community structure (Figure 4A, Supplementary Table 1). SWC, pH, SOC, TN, DOC, TP, AP, and MBC were the important factors contributing to variation in soil bacterial community structure in Ws plots (Figure 4B, Supplementary Table 1). SWC, SOC, DOC, TN, TP, MBC, MBN, and MBP were the important factors contributing to variation in soil bacterial community structure in the Wh plots (Figure 4C, Supplementary Table 1). In contrast, the vertical structure of the fungal communities in all plots was not significantly affected by the soil characters (Figure 3), while plant biomass was an important factor related to the fungal communities in the Ws and Wh plots (Figures 4E,F, Supplementary Table 1). The ecological processes of the microbial communities across the soil layers were inferred by the DNCI. The DNCI values of the bacterial communities were positive in all treatments, and increased in response to temperature in the upper and lower layers and in total, indicating that niche-based ecology processes were important in bacterial communities and the increased relative importance of the niche process was enhanced by warming (Figure 5A). The DNCI values of the fungal communities were negative in the CK plots but increased close to 0 in the Warming plots, indicating that dispersal was the main process of the fungal communities in the CK plots. The relative importance of dispersal decreased and was close to equal to niche process in the warming plots (Figure 5B). We calculated Pearson's correlation coefficients between the DNCI and soil characters, and the DNCI of the bacterial community was significantly correlated with the variation in pH (*r* = 0.747, *p* < 0.05), while the DNCI of the fungal community was significantly negatively correlated with the mean of the SWC (*r* = −0.684, *p* < 0.05), MBC/MBP (*r* = −0.885, *p* < 0.01) and MBN/MBP (*r* = −0.897, *p* < 0.01) (Table 2).

**Table 2:** Pearson's correlation coefficients (r values) between the DNCI of the bacterial and fungi communities with the soil characteristics.

	**Bacteria**		**Fungi**	
	**Variation**	**Mean**	**Variation**	**Mean**
SWC	0.509	−0.343	0.740	−0.684*
pH	0.747*	−0.387	0.533	−0.602
SOC	0.538	0.218	0.254	0.157
TN	0.602	0.244	0.276	0.081
TP	0.628	0.387	0.229	0.483
AP	0.039	0.096	−0.202	0.352
DOC	0.443	0.096	0.183	0.020
${\text{NH}}_{4}^{+}$	0.641	0.101	0.373	−0.047
${\text{NO}}_{3}^{-}$	−0.659	−0.687	−0.286	−0.579
MBC	0.235	−0.303	0.173	−0.371
MBN	0.558	−0.025	0.060	−0.081
MBP	0.543	0.352	0.252	0.581
MBC/MBN	0.497	−0.518	0.526	−0.539
MBC/MBP	0.136	−0.524	0.130	–0.885**
MBN/MBP	0.210	−0.472	0.916	–0.897**

MBC, microbial biomass carbon; MBN, microbial biomass nitrogen; MBP, microbial biomass phosphorous; Variation, the variation of soil characters; Mean, the mean of soil characters.

*0.01 < *p* < 0.05; **0.001 < *p* < 0.01.

## Discussion

### Warming did not affect soil microbial diversity but shifted the fungal community structure

Diversity indices are important indicators of soil microbial diversity. The alpha diversity of the bacterial and fungal communities did not change significantly after the short-term warming treatments in the alpine peatland which support hypothesis I. Previous studies have shown that warming treatments have significant or no effects on microbial communities, which is related to the experimental duration, intensity, frequency of warming, and the availability of substrates for microbial growth (Finlay et al., 1997; Frey et al., 2008; Adair et al., 2019). The same results were reported in alpine grasslands and the cultivated grasslands of the Qinghai-Tibetan Plateau (Zhang Y. et al., 2016). Unlike farmland ecosystems (Sun et al., 2018), the richness of the fungal community was significantly affected by soil depth in this study. Fungi are generally correlated with plants, as they play a critical role in linking below ground with the above ground in the terrestrial ecosystem (Rillig, 2004; Wardle et al., 2004; Hannula and Trager, 2020). The fungal communities could have been affected by roots, as the vegetation type at the study site was herbaceous, which declined with depth.

Proteobacteria and Actinobacteriota were the dominant phyla as reported by a previous study on the Zoige alpine peatland (Fan et al., 2021). The relative abundance of Proteobacteria is also high in moist soils due to its wide adaptability (Jiang et al., 2013; Kang et al., 2021). The bacterial communities did not respond significantly to warming (Figure 3), although several groups in particular soil layers were sensitive to warming (Supplementary Figure 2). The changes in the peatland fungal groups were thought to be related to the magnitude of the temperature increase, as the composition of the fungal communities responded differently to warming at +4 and +8°C (Asemaninejad et al., 2018). In this short-term warming study, the relative abundance of Mortierellomycota was higher under warming at +0.8°C, while it was not significantly different from the control under warming at +1.8°C, indicating that the response of the fungi community to warming was complexed. More experiments with temperature gradients are needed to reveal the mechanisms by which fungal communities respond to warming.

### Warming did not affect the vertical structure of bacterial communities but fungal communities

The majority of the bacterial groups were significantly different among the soil layers, while the fungi groups revealed no distinct vertical patterns. Soil aggregates provide a large number of ecological niches, and the vertical distribution of soil microbial communities that live inside soil aggregates is generally limited by soil environmental factors (Sun et al., 2021). The vertical pattern of the bacterial community is more likely to be correlated with soil C and nutrients (Chu et al., 2016; Du et al., 2017; Sun et al., 2018; Brewer et al., 2019), which was also observed in this study. The structure of the bacterial communities in the warming plots was regulated more strongly by soil moisture and nutrients compared to the CK plots (Figure 4). The microbial response to warming may be related to moisture (Sheik et al., 2011; Peltoniemi et al., 2015). Soil bacteria are the main drivers of peatland carbon cycling, and a decrease in wetland soil moisture may increase soil permeability and thus promote the decomposition of soil nutrients (Ladau et al., 2018). The bacterial communities in the lower and middle soil layers became more similar after the temperature increased. Deterministic processes that drive soil prokaryotic communities increase with depth (Du et al., 2021), making it easier for soil microbes in deeper layers to converge as selection increases. However, the structure of the bacterial communities did not differ between the CK and warming plots (Figure 3A), which might be attributed to the delayed response of bacteria to warming (Ladau et al., 2018), differing from hypothesis II. In contrast, the vertical structure of the fungal community responded significantly to warming (Figure 3B). The structure of the fungal community was not significantly related to environmental factors in the CK plots but was significantly correlated with plant biomass in the warming plots (Figure 4). Considering the changes in the fungi community (Figure 5), warming-induced changes in soil moisture may enhance the niche-based processes of the fungal community, thereby enhancing the interactions between the soil fungal community and plants, which needs to be verified by subsequent studies.

### Warming enhanced the niche process of bacterial assembly and weakened the dispersal process of fungal assembly

**Figure 6:**
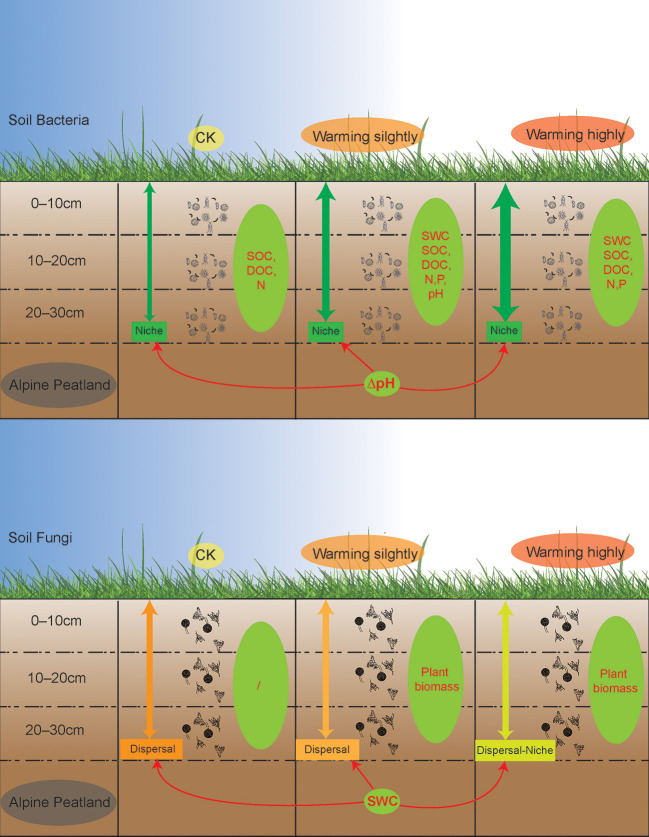
A schematic plot to show the vertical assembly of soil bacterial and fungal communities' response to warming in alpine peatland. Thicker green arrows indicate stronger niche processes while darker orange arrows indicate stronger dispersal processes. Green ellipses represent factors driving the vertical structure of the microbial communities, while the red one-way arrows represent factors that influence the vertical assembly of the community.

Disentangling ecological processes controlling community assembly is crucial in microbial ecology (Zhou and Ning, 2017). In this study, we focused on the vertical distribution of the soil microbial communities across soil layers. The DNCI index was used to clarify the assembly process of the soil microbial community across soil layers. The results showed that the vertical distribution of the soil microbial community was regulated by the niche process, and the niche process was enhanced by warming (Figure 6). Experiments simulating environmental change have shown that changes in soil bacterial community assembly are usually generated by promoting or inhibiting random processes (Zhang X. et al., 2016). The correlation between the DNCI and environmental factors showed that pH was significantly positively correlated with the niche-based process of soil bacteria, which was similar to the results of Luan et al. (2020). Soil pH is an important factor mediating the balance between the stochastic and deterministic assembly of bacteria in successional soils (Tripathi et al., 2018).

The niche-based process is more important for the bacterial than the fungal community in structuring their vertical distribution (Sun et al., 2018). In this study, the fungal communities were mainly regulated by a dispersal-based process, and warming weakened the effect of random dispersal (Figure 6). The Mantel test indicated no strong correlation between the fungal community structure and soil factors. The results of an alpine meadows study showed that 3 years of warming enhances the deterministic processes of the fungal community (Xu et al., 2022). Unexpectedly, a decrease in the relative importance of random processes of the fungal communities was observed after one growing season of warming in our study, indicating that the fungal communities in alpine peatlands are highly sensitive to temperature change. SWC was correlated with the DNCI, suggesting that the dispersal ability of the fungal communities across soil layers was weakened due to reduced moisture in wet soil. Notably, as the DNCI is calculated based on occurrence data, the potential mechanism driven by the abundance changes may be underestimated (Vilmi et al., 2020).

## Conclusion

We investigated the effects on vertical distribution and assembly of soil microbes under one growing season of warming in an alpine peatland. We found that short-term warming had no significant effects on the alpha diversity of either the bacteria or the fungi but altered the structure of fungi community. The vertical pattern of the fungal community in the alpine peatland was sensitive to warming. The vertical assembly of the fungal community was affected by soil moisture during short-term warming, while the relative importance of the niche-based process for bacteria increased with the variation in soil pH. Our results could provide a potential mechanism of microbial community vertical assembly in the alpine peatland in response to warming. Long-term warming and integrative studies are needed to clarify the distinction between the vertical and horizontal distributions and the assembly of soil microbial communities in the future.

## Data availability statement

The data presented in the study are deposited in the NCBI BioProject under accession number (PRJNA862351).

## Author contributions

XW, YL, YH, EK, and XK contributed to the conception and the design of the study. XW, EK, YN, and AY conducted the experiments. XW and YL performed the statistical analysis. XW and XK wrote the first draft of the manuscript. XZ, ZY, ML, LY, and KZ wrote sections of the manuscript. All the authors contributed to the manuscript revision and read and approved the submitted version.

## Funding

This study was supported by the National Natural Science Foundation of China (No. 42041005), the Second Tibetan Plateau Scientific Expedition and Research Program (STEP) Grant (No. 2019 QZKK0304), and the National Natural Science Foundation of China (32171597, 32171598).

## Conflict of interest

The authors declare that the research was conducted in the absence of any commercial or financial relationships that could be construed as a potential conflict of interest.

## Publisher's note

All claims expressed in this article are solely those of the authors and do not necessarily represent those of their affiliated organizations, or those of the publisher, the editors and the reviewers. Any product that may be evaluated in this article, or claim that may be made by its manufacturer, is not guaranteed or endorsed by the publisher.
