# Supplementary material for: The divergent vertical pattern and assembly of soil bacterial and fungal communities in response to short-term warming in an alpine peatland
Source: Front Plant Sci. 2022 Sep 8;13:986034. doi: 10.3389/fpls.2022.986034 (PMC9493461; doi:10.3389/fpls.2022.986034)
Supplement: Supplementary file 1 [file Data_Sheet_1.docx]

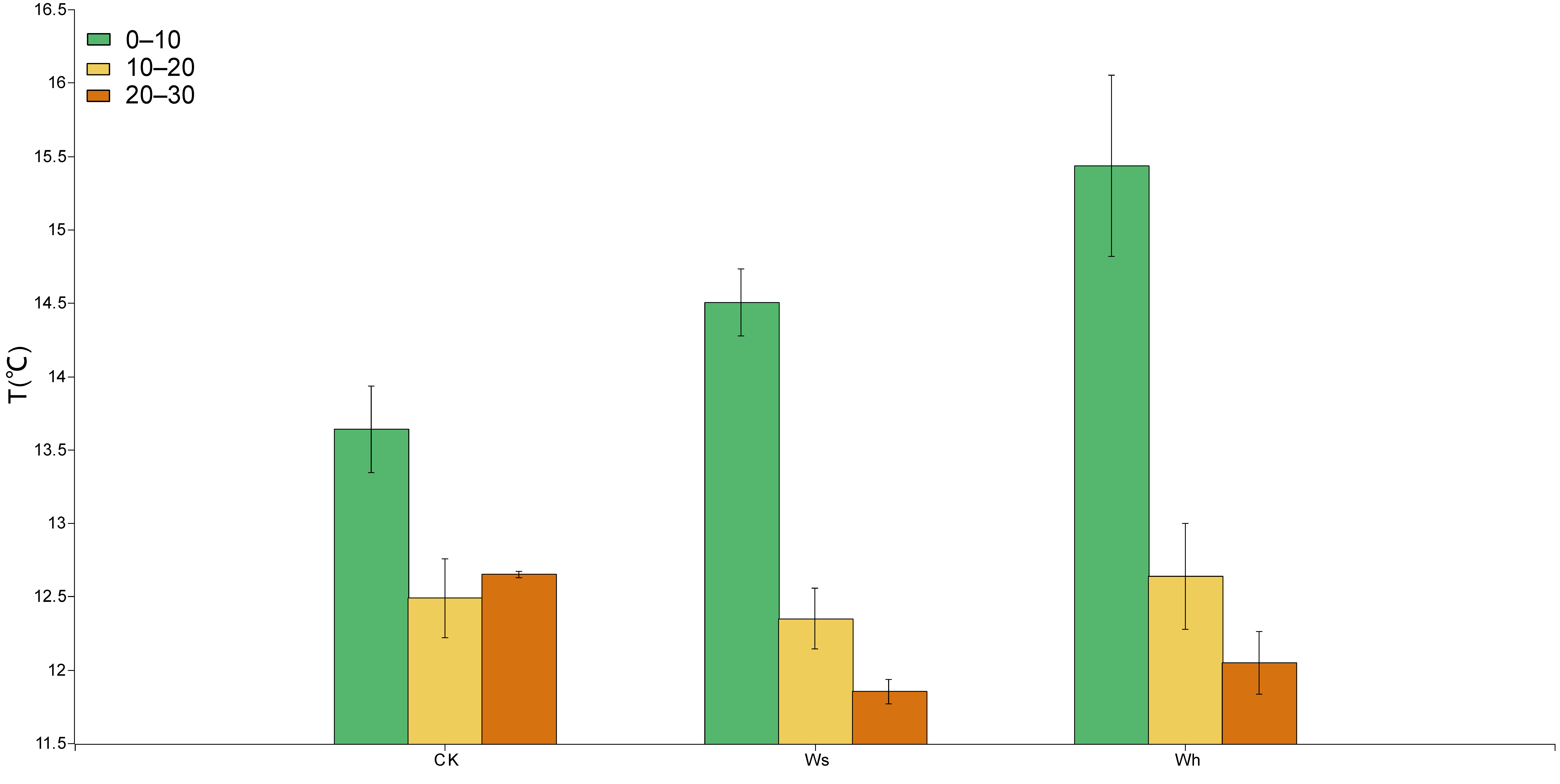


**Figure 1** Average temperature of soil layers during the growing season (measured every ten days). Ws, slight warming; Wh, high warming.


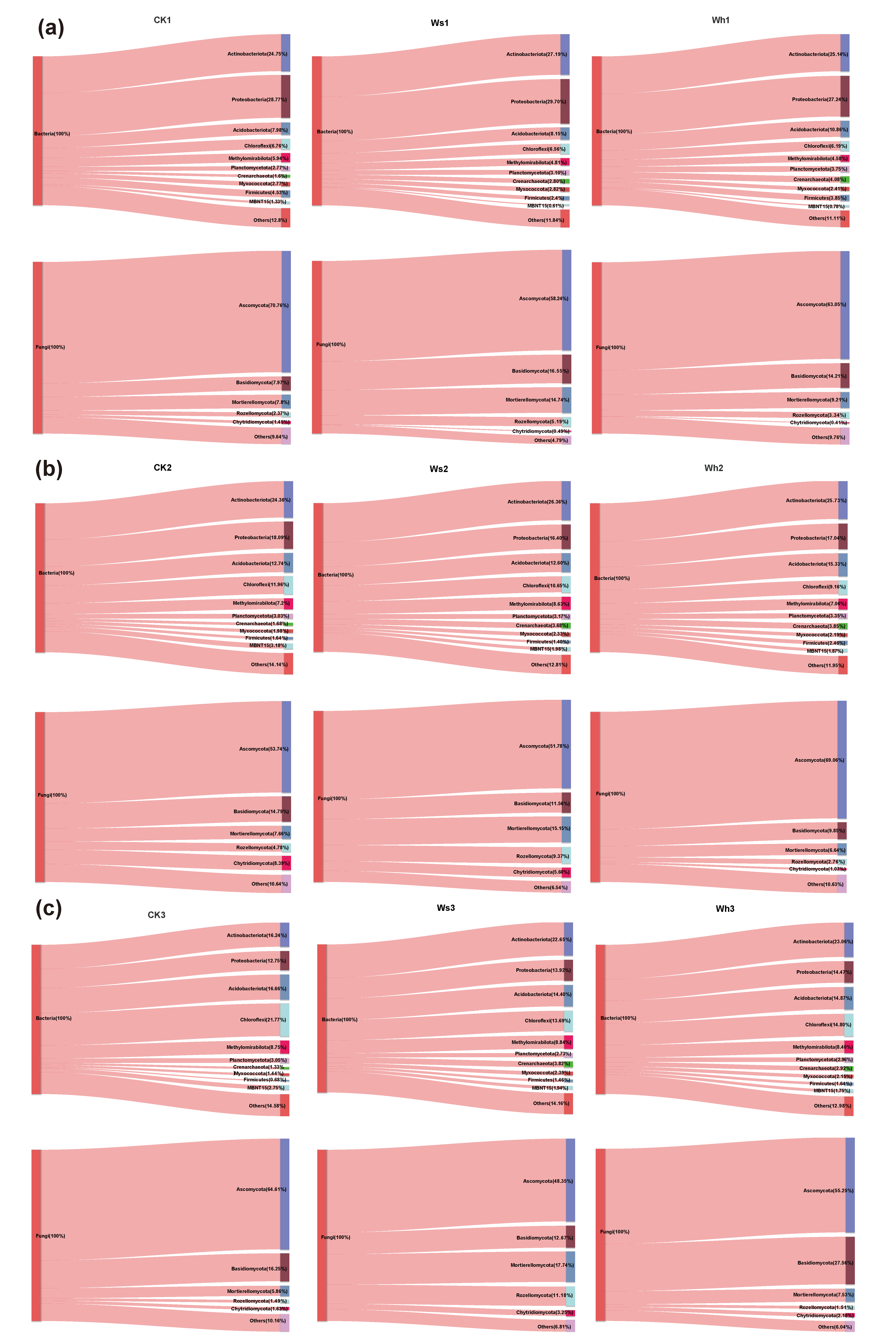


**Figure 2** A sankeymatic diagram of composition of bacterial and fungal communities at phylum level. Ws, slight warming; Wh, high warming. CK1, 0–10 cm depth in control plots; CK2, 10–20 cm depth in control plots; CK3, 20–30 cm depth in control plots.


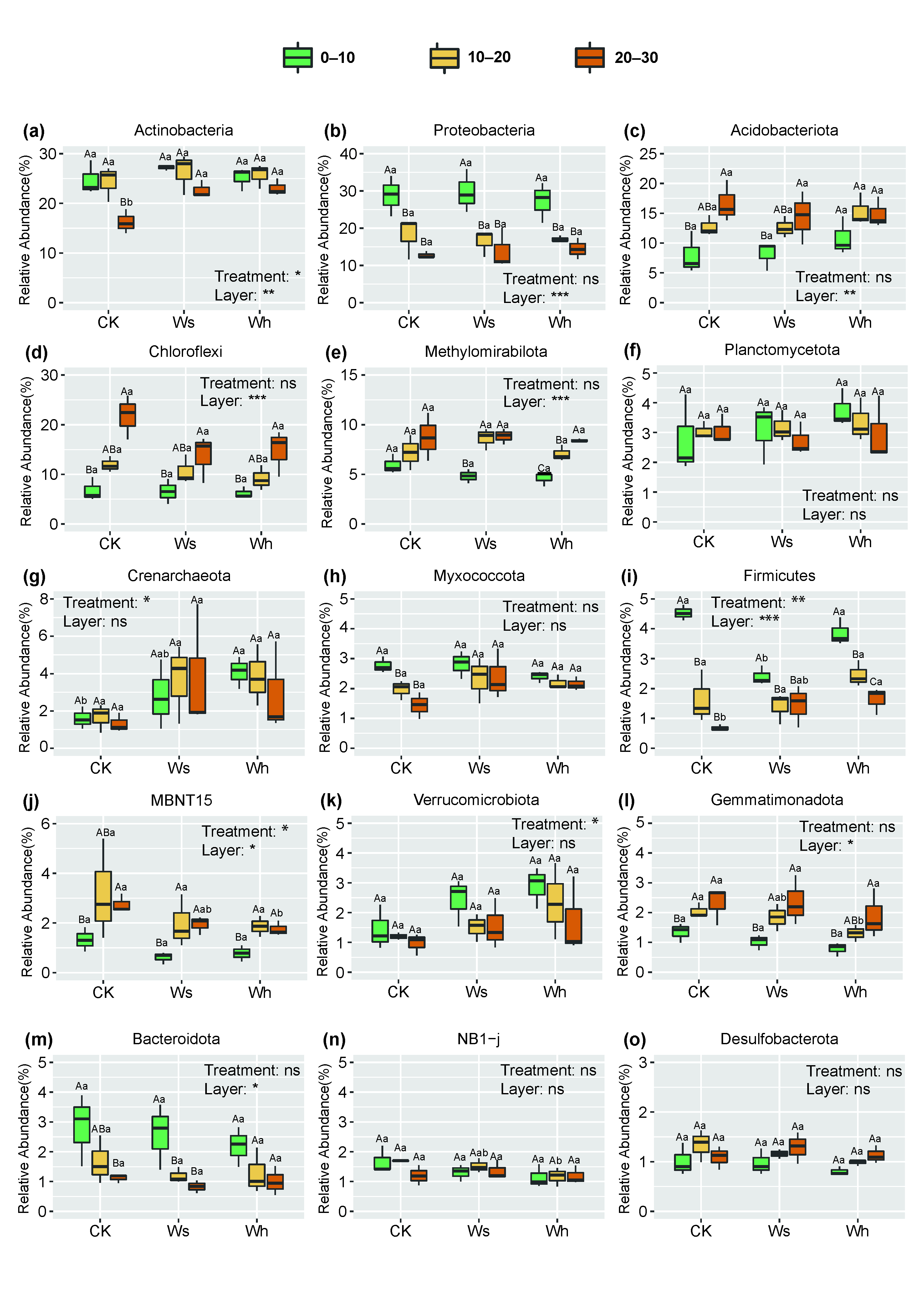
**Figure 3** Relative abundance of different phyla of soil bacteria. Only phyla with greater than 1% representation are shown. “*”, “**”, “***”, and “ns” indicate the significant levels for treatments and soil layers (nested within treatments) at 0.05, 0.01, 0.001, and non-significant, respectively. Boxplots not sharing a common [capital](javascript:;) letter are significantly different (*p* < 0.05) among soil layers while different small letters represent significantly different (*p* < 0.05) among treatments. Ws, slight warming; Wh, high warming.


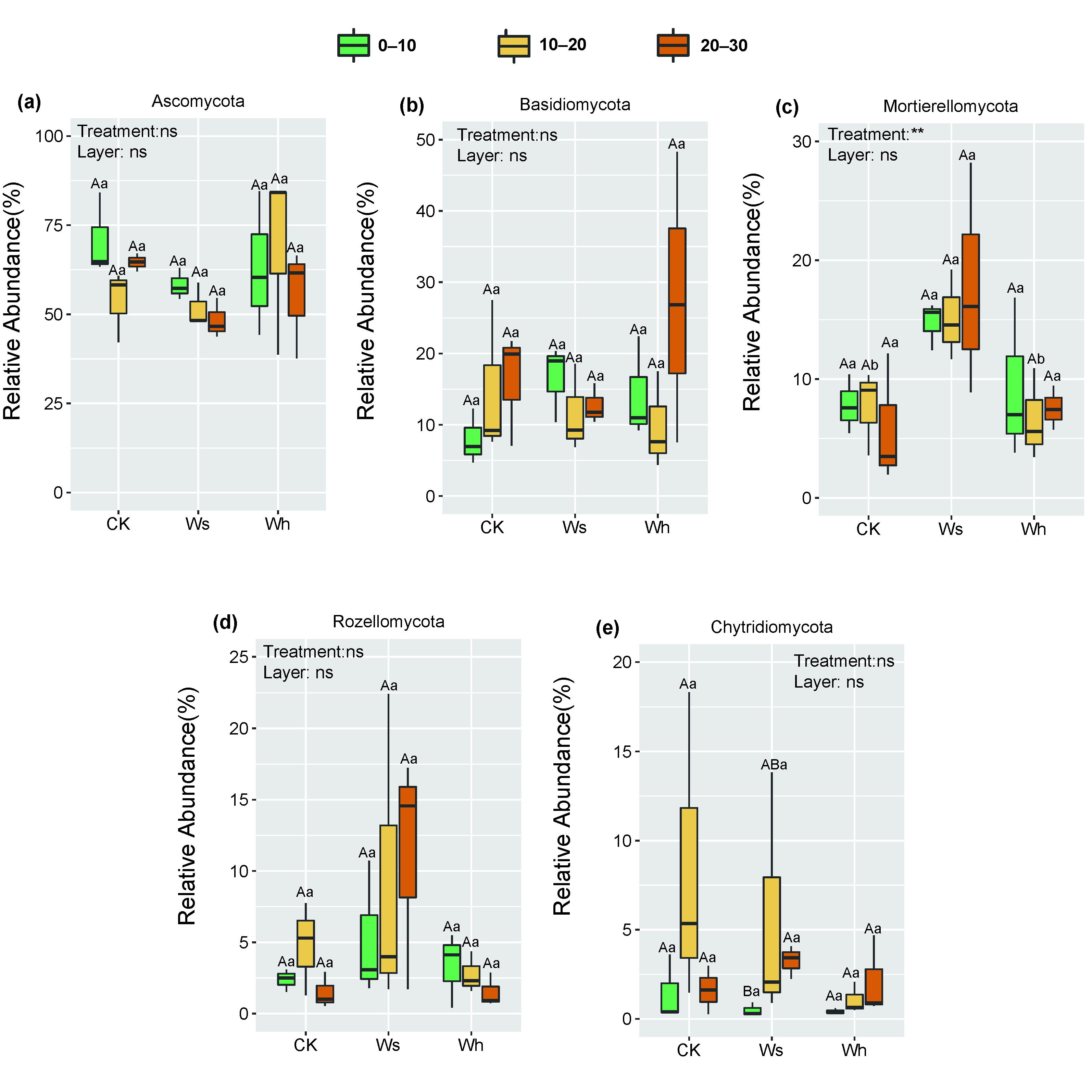
**Figure 4** Relative abundance of different phyla of soil fungi. Only phyla with greater than 1% representation are shown. “*”, “**”, “***”, and “ns” indicate the significant levels for treatments and soil layers (nested within treatments) at 0.05, 0.01, 0.001, and non-significant, respectively. Boxplots not sharing a common [capital](javascript:;) letter are significantly different (*p* < 0.05) among soil layers while different small letters represent significantly different (*p* < 0.05) among treatments. Ws, slight warming; Wh, high warming.


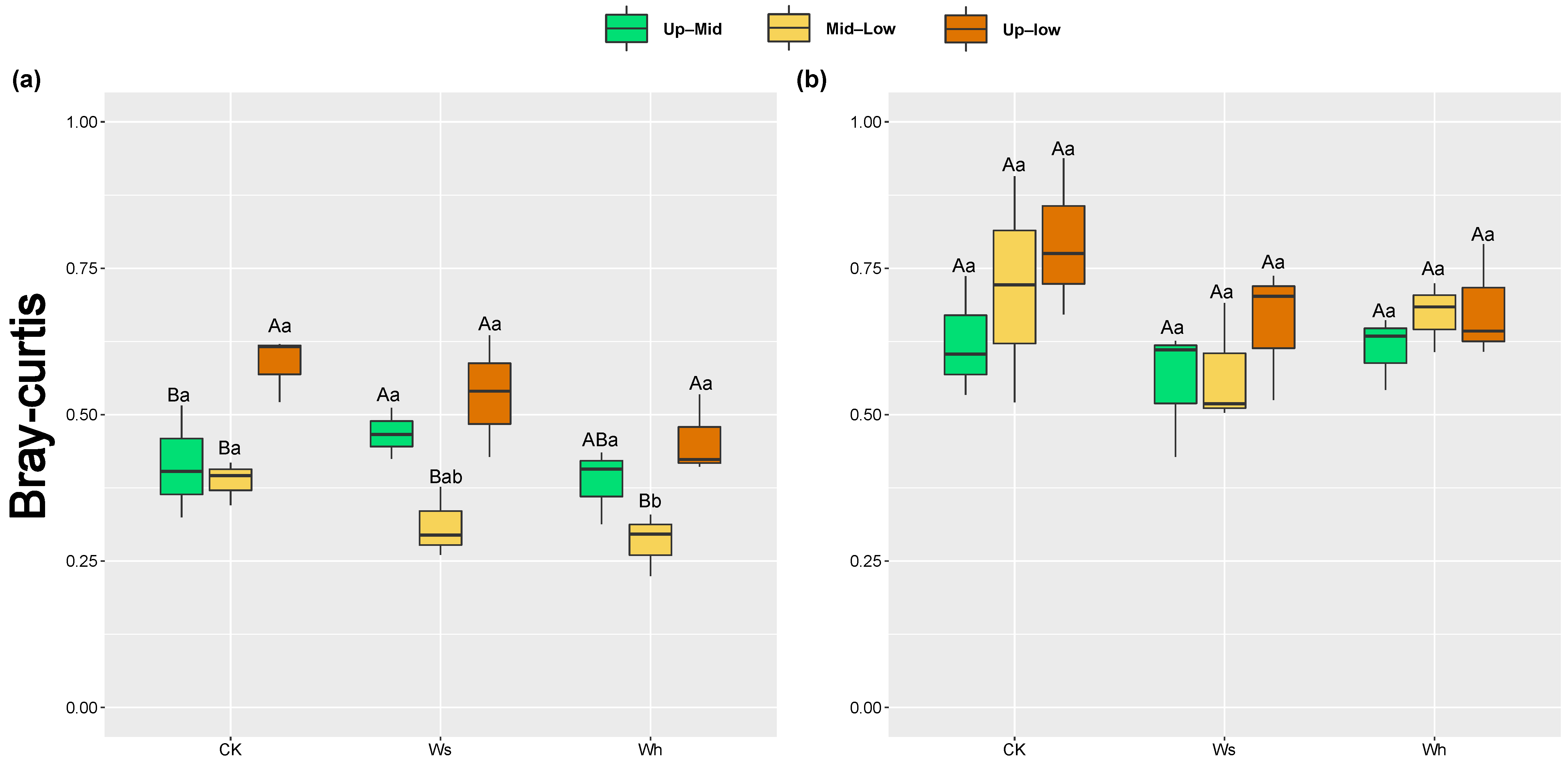


**Figure 5** Bray-Curtis dissimilarity index of soil bacterial and fungal communities. Boxplots not sharing a common [capital](javascript:;) letter are significantly different (*p* < 0.05) among soil layers while different small letters represent significantly different (*p* < 0.05) among treatments. Ws, slight warming; Wh, high warming; Wh, warming highly; Up, 0–10 cm soil layer; Mid, 10–20 cm soil layer; Low, 20–30 cm soil layer.

**Table 1** Results of mantel test (r value) between community structure of soil bacterial and fungal with soil characteristics and plant biomass.

|  | CK | | Ws | | Wh | |
| --- | --- | --- | --- | --- | --- | --- |
|  | r | p | r | p | r | p |
| Bacteria | | | | | | |
| SWC | 0.368 | **0.048*** | 0.560 | **0.009**** | 0.596 | **0.004**** |
| pH | -0.004 | 0.505 | 0.609 | **0.005**** | 0.142 | 0.285 |
| SOC | 0.692 | **0.004**** | 0.655 | **0.002**** | 0.745 | **0.001***** |
| TN | 0.386 | **0.030*** | 0.653 | **0.001***** | 0.596 | **0.009**** |
| TP | 0.252 | 0.077 | 0.374 | **0.042*** | 0.832 | **0.002**** |
| AP | 0.384 | **0.042*** | 0.410 | **0.022*** | 0.334 | 0.064 |
| DOC | 0.644 | **0.005**** | 0.668 | **0.001***** | 0.776 | **0.001***** |
| NH_4_^+^ | 0.057 | 0.304 | 0.215 | 0.105 | 0.202 | 0.178 |
| NO_3_^-^ | 0.612 | **0.002**** | -0.226 | 0.796 | 0.039 | 0.408 |
| MBC | 0.410 | **0.027*** | 0.482 | **0.020*** | 0.556 | **0.009**** |
| MBN | 0.459 | **0.017*** | 0.278 | 0.066 | 0.711 | **0.002**** |
| MBP | 0.410 | **0.028*** | 0.168 | 0.157 | 0.405 | **0.020*** |
| C/N | 0.155 | 0.180 | -0.300 | 0.951 | 0.127 | 0.297 |
| C/P | 0.226 | 0.099 | -0.123 | 0.721 | 0.077 | 0.370 |
| N/P | 0.126 | 0.169 | 0.109 | 0.257 | -0.029 | 0.520 |
| Biomass | -0.018 | 0.431 | 0.055 | 0.342 | 0.201 | 0.096 |
|  | | | | | | |
| Fungi | | | | | | |
| SWC | -0.096 | 0.640 | 0.224 | 0.097 | 0.125 | 0.221 |
| pH | -0.185 | 0.788 | 0.094 | 0.344 | 0.048 | 0.309 |
| SOC | 0.011 | 0.462 | -0.010 | 0.543 | -0.062 | 0.600 |
| TN | 0.010 | 0.401 | 0.149 | 0.197 | -0.048 | 0.591 |
| TP | -0.162 | 0.684 | 0.243 | 0.089 | 0.093 | 0.289 |
| AP | -0.131 | 0.715 | -0.192 | 0.806 | 0.125 | 0.209 |
| DOC | 0.074 | 0.316 | -0.028 | 0.527 | 0.076 | 0.303 |
| NH_4_^+^ | -0.136 | 0.694 | 0.201 | 0.182 | -0.151 | 0.815 |
| NO_3_^-^ | 0.082 | 0.323 | -0.158 | 0.726 | 0.251 | 0.090 |
| MBC | -0.169 | 0.838 | 0.209 | 0.105 | 0.224 | 0.101 |
| MBN | 0.086 | 0.320 | 0.303 | 0.069 | 0.124 | 0.215 |
| MBP | -0.167 | 0.838 | 0.274 | 0.090 | -0.089 | 0.690 |
| C/N | 0.630 | 0.067 | -0.222 | 0.829 | -0.211 | 0.941 |
| C/P | -0.115 | 0.679 | -0.081 | 0.630 | -0.180 | 0.869 |
| N/P | -0.266 | 0.952 | 0.158 | 0.216 | -0.164 | 0.872 |
| Biomass | 0.237 | 0.080 | 0.629 | **0.001***** | 0.384 | **0.027**** |

Ws, slight warming; Wh, high warming; MBC, microbial biomass carbon; MBN, microbial biomass nitrogen; MBP, microbial biomass phosphorous; C/N, MBC/MBN; C/P, MBC/MBP; N/P, MBN/MBP; Biomass, plant biomass. *, 0.01< p < 0.05; **, 0.01< p < 0.05; ***, p < 0.001.

**Table 2** Results of PERMANOVA (adonis) on the composition of Bacterial and Fungal communities.

|  |  | Bacteria | | | | Fungi | | | |
| --- | --- | --- | --- | --- | --- | --- | --- | --- | --- |
|  | Groups | F.Model | R^2^ | p.value | p.adjusted | F.Model | R^2^ | p.value | p.adjusted |
| 0-10 | CK vs Ws | 1.938 | 0.326 | 0.100 | 0.300 | 1.513 | 0.274 | 0.100 | 0.300 |
|  | CK vs Wh | 1.404 | 0.260 | 0.300 | 0.900 | 1.167 | 0.226 | 0.200 | 0.600 |
|  | Ws vs Wh | 1.080 | 0.213 | 0.200 | 0.600 | 0.943 | 0.191 | 0.800 | 1.000 |
| 10-20 | CK vs Ws | 0.903 | 0.184 | 0.600 | 1.000 | 1.440 | 0.265 | 0.100 | 0.300 |
|  | CK vs Wh | 1.302 | 0.246 | 0.200 | 0.600 | 1.390 | 0.258 | 0.200 | 0.600 |
|  | Ws vs Wh | 0.762 | 0.160 | 0.600 | 1.000 | 1.548 | 0.279 | 0.100 | 0.300 |
| 20-30 | CK vs Ws | 1.792 | 0.309 | 0.100 | 0.300 | 1.324 | 0.249 | 0.100 | 0.300 |
|  | CK vs Wh | 2.249 | 0.360 | 0.100 | 0.300 | 1.014 | 0.202 | 0.600 | 1.000 |
|  | Ws vs Wh | 0.457 | 0.103 | 0.900 | 1.000 | 1.201 | 0.231 | 0.200 | 0.600 |
| ALL-layers | CK vs Ws | 1.458 | 0.084 | 0.176 | 0.528 | 2.936 | 0.155 | 0.001 | **0.003**** |
|  | CK vs Wh | 1.897 | 0.106 | 0.096 | 0.288 | 2.166 | 0.119 | 0.001 | **0.003**** |
|  | Ws vs Wh | 0.731 | 0.044 | 0.550 | 1.000 | 2.398 | 0.130 | 0.001 | **0.003**** |
| CK | 0-10 vs 10-20 | 3.251 | 0.448 | 0.100 | 0.300 | 0.675 | 0.144 | 0.900 | 1.000 |
|  | 0-10 vs 20-30 | 7.587 | 0.655 | 0.100 | 0.300 | 1.091 | 0.214 | 0.300 | 0.900 |
|  | 10-20 vs 20-30 | 1.986 | 0.332 | 0.100 | 0.300 | 0.748 | 0.158 | 0.900 | 1.000 |
| Ws | 0-10 vs 10-20 | 4.039 | 0.502 | 0.100 | 0.300 | 0.815 | 0.169 | 0.700 | 1.000 |
|  | 0-10 vs 20-30 | 4.874 | 0.549 | 0.100 | 0.300 | 1.034 | 0.205 | 0.400 | 1.000 |
|  | 10-20 vs 20-30 | 0.661 | 0.142 | 0.800 | 1.000 | 0.737 | 0.156 | 0.800 | 1.000 |
| Wh | 0-10 vs 10-20 | 3.462 | 0.464 | 0.100 | 0.300 | 0.946 | 0.191 | 0.500 | 1.000 |
|  | 0-10 vs 20-30 | 4.347 | 0.521 | 0.100 | 0.300 | 0.674 | 0.144 | 0.900 | 1.000 |
|  | 10-20 vs 20-30 | 1.162 | 0.225 | 0.300 | 0.900 | 0.963 | 0.194 | 0.400 | 1.000 |
| ALL-treat | 0-10 vs 10-20 | 8.907 | 0.358 | 0.001 | **0.003**** | 0.959 | 0.057 | 0.554 | 1.000 |
|  | 0-10 vs 20-30 | 12.723 | 0.443 | 0.001 | **0.003**** | 1.370 | 0.079 | 0.063 | 0.189 |
|  | 10-20 vs 20-30 | 2.332 | 0.127 | 0.031 | 0.093 | 0.896 | 0.053 | 0.658 | 1.000 |

Ws, slight warming; Wh, high warming; *, 0.01< p < 0.05; **, 0.01< p < 0.05; ***, p < 0.001.

**Table 3** Results of ANOSIM on the composition of Bacterial and Fungal communities.

|  |  | Bacteria | | | Fungi | | |
| --- | --- | --- | --- | --- | --- | --- | --- |
|  | Groups | R | p.value | p.adjusted | R | p.value | p.adjusted |
| 0-10 | CK vs Ws | 0.667 | 0.100 | 0.157 | 0.667 | 0.100 | 0.327 |
|  | CK vs Wh | 0.111 | 0.400 | 0.533 | 0.111 | 0.500 | 0.667 |
|  | Ws vs Wh | -0.037 | 0.600 | 0.635 | -0.074 | 0.900 | 0.926 |
| 10-20 | CK vs Ws | -0.111 | 0.500 | 0.600 | 0.370 | 0.100 | 0.327 |
|  | CK vs Wh | 0.074 | 0.600 | 0.635 | 0.259 | 0.200 | 0.400 |
|  | Ws vs Wh | -0.111 | 0.500 | 0.600 | 0.296 | 0.300 | 0.470 |
| 20-30 | CK vs Ws | 0.296 | 0.200 | 0.288 | 0.333 | 0.100 | 0.327 |
|  | CK vs Wh | 0.519 | 0.100 | 0.157 | 0.000 | 0.600 | 0.720 |
|  | Ws vs Wh | -0.296 | 0.900 | 0.900 | 0.185 | 0.200 | 0.400 |
| ALL-layers | CK vs Ws | 0.049 | 0.215 | 0.323 | 0.469 | 0.001 | **0.001***** |
|  | CK vs Wh | 0.118 | 0.095 | 0.285 | 0.283 | 0.001 | **0.001***** |
|  | Ws vs Wh | -0.020 | 0.474 | 0.474 | 0.346 | 0.001 | **0.001***** |
| CK | 0-10 vs 10-20 | 0.778 | 0.100 | 0.157 | -0.333 | 1.000 | 1.000 |
|  | 0-10 vs 20-30 | 1.000 | 0.100 | 0.157 | 0.074 | 0.400 | 0.554 |
|  | 10-20 vs 20-30 | 0.593 | 0.100 | 0.157 | -0.074 | 0.800 | 0.873 |
| Ws | 0-10 vs 10-20 | 0.778 | 0.100 | 0.157 | -0.074 | 0.700 | 0.813 |
|  | 0-10 vs 20-30 | 0.852 | 0.100 | 0.157 | 0.148 | 0.300 | 0.470 |
|  | 10-20 vs 20-30 | -0.148 | 0.900 | 0.900 | -0.074 | 0.600 | 0.720 |
| Wh | 0-10 vs 10-20 | 0.741 | 0.100 | 0.157 | 0.000 | 0.600 | 0.720 |
|  | 0-10 vs 20-30 | 0.815 | 0.100 | 0.157 | -0.296 | 0.900 | 0.926 |
|  | 10-20 vs 20-30 | 0.037 | 0.400 | 0.533 | -0.148 | 0.800 | 0.873 |
| ALL-treat | 0-10 vs 10-20 | 0.763 | 0.001 | **0.002**** | 0.002 | 0.438 | 0.531 |
|  | 0-10 vs 20-30 | 0.842 | 0.001 | **0.002**** | 0.106 | 0.046 | 0.138 |
|  | 10-20 vs 20-30 | 0.182 | 0.030 | 0.030 | -0.011 | 0.531 | 0.531 |

Ws, slight warming; Wh, high warming; *, 0.01< p < 0.05; **, 0.01< p < 0.05; ***, p < 0.001.
